# Feasibility of Implanting a Foot–Ankle Endoprosthesis within Skin in a Rabbit Model of Transtibial Amputation

**DOI:** 10.3390/bioengineering9080348

**Published:** 2022-07-27

**Authors:** Dustin L. Crouch, Patrick T. Hall, Caleb Stubbs, Caroline Billings, Alisha P. Pedersen, Bryce Burton, Cheryl B. Greenacre, Stacy M. Stephenson, David E. Anderson

**Affiliations:** 1 Department of Mechanical, Aerospace & Biomedical Engineering, College of Engineering, University of Tennessee, Knoxville, TN 37996, USA; phall@exponent.com (P.T.H.); rstubbs1@vols.utk.edu (C.S.); 2 Exponent, Philadelphia, PA 19104, USA; 3 Department of Large Animal Clinical Sciences, College of Veterinary Medicine, University of Tennessee, Knoxville, TN 37996, USA; cbilli10@vols.utk.edu (C.B.); apotte14@vols.utk.edu (A.P.P.); dander48@utk.edu (D.E.A.); 4 Office of Laboratory Animal Care, College of Veterinary Medicine, University of Tennessee, Knoxville, TN 37996, USA; bburto11@utk.edu; 5 Department of Small Animal Clinical Sciences, College of Veterinary Medicine, University of Tennessee, Knoxville, TN 37996, USA; cgreenac@utk.edu; 6 Graduate School of Medicine, University of Tennessee, Knoxville, TN 37920, USA; sstephenson@utmck.edu

**Keywords:** implant, orthopedic, skin, device, rabbit, animal, silicone, hindlimb

## Abstract

Prosthetic limbs that are completely implanted within skin (i.e., endoprostheses) could permit direct, physical muscle–prosthesis attachment to restore more natural sensorimotor function to people with amputation. The objective of our study was to test, in a rabbit model, the feasibility of replacing the lost foot after hindlimb transtibial amputation by implanting a novel rigid foot–ankle endoprosthesis that is fully covered with skin. We first conducted a pilot, non-survival surgery in two rabbits to determine the maximum size of the skin flap that could be made from the biological foot–ankle. The skin flap size was used to determine the dimensions of the endoprosthesis foot segment. Rigid foot–ankle endoprosthesis prototypes were successfully implanted in three rabbits. The skin incisions healed over a period of approximately 1 month after surgery, with extensive fur regrowth by the pre-defined study endpoint of approximately 2 months post surgery. Upon gross inspection, the skin surrounding the endoprosthesis appeared normal, but a substantial subdermal fibrous capsule had formed around the endoprosthesis. Histology indicated that the structure and thickness of the skin layers (epidermis and dermis) were similar between the operated and non-operated limbs. A layer of subdermal connective tissue representing the fibrous capsule surrounded the endoprosthesis. In the operated limb of one rabbit, the subdermal connective tissue layer was approximately twice as thick as the skin on the medial (skin = 0.43 mm, subdermal = 0.84 mm), ventral (skin = 0.80 mm, subdermal = 1.47 mm), and lateral (skin = 0.76 mm, subdermal = 1.42 mm) aspects of the endoprosthesis. Our results successfully demonstrated the feasibility of implanting a fully skin-covered rigid foot–ankle endoprosthesis to replace the lost tibia–foot segment of the lower limb. Concerns include the fibrotic capsule which could limit the range of motion of jointed endoprostheses. Future studies include testing of endoprosthetics, as well as materials and pharmacologic agents that may suppress fibrous encapsulation.

## 1. Introduction

The physical connection between muscles and limb segments across moving joints is a key feature that enables neuromuscular control and sensation of movement in biological limbs. Limb amputation severs the physical connection between muscles and limb segments, resulting in complete loss of neuromuscular function associated with the missing limb. Functional limb prostheses are commonly prescribed to replace part of the missing limb’s neuromuscular function. However, all existing prostheses must be worn externally, limiting the extent to which they can directly interface with the neuromuscular system. Specifically, muscles, the biological motors, are typically bypassed in favor of electromechanical systems (e.g., motors, sensors, computers) to generate movements [1,2,3,4,5,6,7,8,9,10,11,12,13]. Inherent limitations of the user–machine interface often result in erroneous movements of external electromechanical prostheses in real-world scenarios [14,15], and movements do not, as of yet, appear natural.

One alternate neuromuscular interface concept seeks to physically attach muscles—the residual muscles that once attached to the missing limb—to prostheses. Such a physical muscle–prosthesis connection may replicate the neuromuscular control and sensation of movement in biological limbs. Since all existing limb prostheses must be worn externally, the previous approach to physically attach muscles to prostheses was achieved through cineplasty surgery [16,17]. Cineplasty connects muscle–tendon units to skin loops so that muscle forces can be transferred through skin to prostheses via cables [17]. Users noted that cineplasty enabled more precise control of prosthesis movements [17,18]. Unfortunately, cineplasty has not been widely adopted due to limitations in function, comfort, and appearance. 

To better facilitate physical muscle–prosthesis attachment and overcome limitations of cineplasty, we propose to implant limb prostheses completely inside the body, fully enclosed within a skin envelope. Compared to cineplasty, an implanted limb prosthesis, or endoprosthesis, would permit muscle attachment to prosthetic limb segments in a more anatomically realistic way. Our current conceptual framework is that endoprostheses would reconstruct part of or all the skeletal anatomy, including joints, of the missing limb; residual muscles would be attached to the endoprosthesis using either biological or artificial tendons [19]. Similar endoprostheses have been used for extensive skeletal reconstruction following tumor resection but are typically implanted within skin and muscles and serve as a bridge between intact biological limb segments [20,21]. Conversely, our proposed endoprosthetic limb would be in direct contact with skin over most of its surface and extend distally from the distal end of a residual limb.

A major unique, anticipated challenge to implanting our proposed endoprostheses in vivo is covering them in living skin at the distal end of a residual limb without interruption of cutaneous blood flow, which is required for nutrient exchange, temperature regulation, and wound healing [22,23]. As the endoprosthesis is made of bioinert materials, the overlying skin can only rely on superficial proximal cutaneous vessels without supplement from deep regional arteries [24]. In a previous study, we successfully implanted a 2-cm long endoprosthesis stem (no foot segment) within skin at the distal end of the residual tibia in rabbits after transtibial amputation [25]. However, to test our muscle-driven endoprosthesis concept, we plan to implant larger endoprostheses with an ankle joint and foot segment in our rabbit model. The feasibility of implanting the larger endoprosthesis, which will require a larger skin flap, is unknown.

The primary objective of the study reported here was to test the feasibility of implanting a foot–ankle endoprosthesis within skin in a rabbit model of hindlimb amputation. This study built on our previous study [25] by adding a 3-cm-long foot segment to the 2-cm-long stem. The ankle joint was rigid (i.e., locked) to reduce the complexity of the endoprosthesis, since the focus of the study was on skin coverage. As in our previous study, we determined feasibility by whether, up to 60 days post surgery, (1) the sutured skin incision fully healed and closed and (2) skin integrity was maintained. A secondary objective was to preliminarily assess the structure and composition of the tissue surrounding the endoprosthesis. 

## 2. Materials and Methods

**Figure 1:**
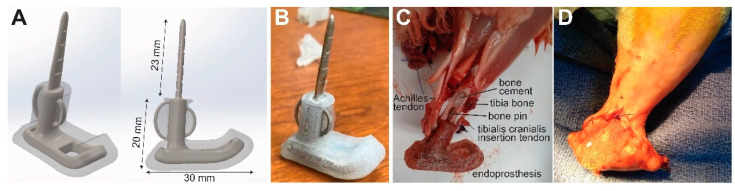
(**A**) Computer-aided design (CAD) model of the endoprosthesis. (**B**) Rigid foot–ankle endoprosthesis prototype fabricated with a 316L stainless steel core and a silicone cover. (**C**) Endoprosthesis in situ in rabbit E0, postmortem. (**D**) Skin flap closed over endoprosthesis in vivo in rabbit E1 immediately after surgery.

We performed two studies, a pilot study and a feasibility study, in sequence. In the pilot study, two healthy, standard, laboratory New Zealand White rabbits (N1, N2) were used in a non-survival surgery to qualitatively assess blood perfusion in skin flaps intended for covering a foot–ankle endoprosthesis prototype; the rabbits were about 34 weeks old and weighed 4.19 ± 0.01 kg at the time of surgery (Table 1). In the subsequent feasibility study, we implanted rigid foot–ankle endoprosthesis prototypes (Figure 1) in four New Zealand White rabbits (E0–E3); the rabbits were approximately 18 weeks old and weighed 3.55 ± 0.53 kg at the time of surgery. Animals were included in the study if they were healthy. As the studies were iterative and not hypothesis-driven, no power analysis was performed. Since this was not a clinical trial, there were no separate experimental groups. Each rabbit was considered one experimental unit. Rabbits were housed individually in adjacent crates, fed ad libitum with a standard laboratory diet and Timothy hay, and given daily enrichment and positive human interaction. All rabbits underwent surgery as described below; however, rabbit E0 died less than an hour after surgery as a result of suspected complications from anesthesia. Potential confounders (e.g., animal/cage location) were not controlled. 

At the beginning of all surgeries described below, each rabbit was sedated, induced into general anesthesia, and maintained by 3–5% isoflurane gas vaporized in 100% O_2_. Either both hind limbs (N1, N2) or only the left hind limb (E0–E3) were clipped and aseptically prepared for surgery. The rabbit was then positioned in lateral recumbency for surgery.

### 2.1. Pilot Study: Non-Survival Surgery to Assess Blood-Perfusion in Skin Flaps

Before fabricating the foot–ankle endoprosthesis prototypes, we performed a pilot study to determine the maximum dimensions for the design of the rigid foot–ankle endoprosthesis. One factor limiting the prototype size was the size and shape of the skin flap used to cover the endoprosthesis. Flap size and shape were limited by the anatomy of the foot and the extent to which blood perfusion could be maintained throughout the skin flap. Bloop perfusion is essential for preventing skin necrosis and ensuring adequate healing along the sutured, apposed edges of the skin flap once it is sutured closed over the endoprosthesis. 

**Figure 2:**
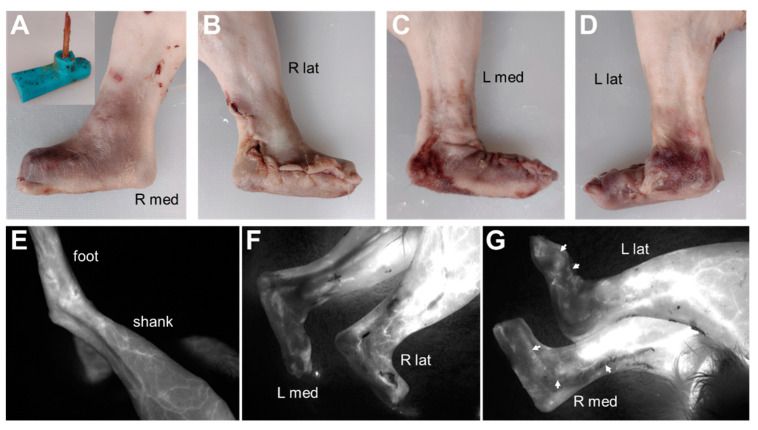
Fluorescence imaging in rabbit N2 to visualize blood flow in skin flap covering a mock foot–ankle prosthesis (**A**, **inset**). (**A**,**B**) Right hindlimb with lateral longitudinal incision. (**C**,**D**) Left hindlimb with cranial longitudinal incision. (**E**) Fluorescence imaging of left hindlimb before incisions. (**F**,**G**) Fluorescence imaging of hindlimbs after skin flaps were sutured closed over the mock prostheses. Blood flow in the skin flap was diminished compared to the pre-incision foot but still visible throughout the skin flap, including along the incision lines (**G**, **arrows**). Qualitatively, blood flow appeared better in the right hindlimb with the lateral longitudinal incision than in the left hindlimb with the cranial longitudinal incision.

Two rabbits, N1 and N2, underwent a non-survival surgery in which we used fluorescein imaging to qualitatively assess blood perfusion in skin flaps formed from the skin surrounding the biological ankle and foot. Both hindlimbs were used in both rabbits. The rabbits were first premedicated with hydromorphone, midazolam, and lidocaine; sedated; induced into general anesthesia; and maintained by isofluorane gas vaporized in 100% O_2_. Using a #10 scalpel blade, a longitudinal skin incision was made from approximately the mid-diaphysis of the tibia to the metatarsophalangeal joint; the longitudinal incision was along the cranial aspect of the hindlimb in the first hindlimb (N1), then along the lateral aspect of the hindlimb in the next three hindlimbs. Qualitatively, the lateral incision better preserved blood perfusion in the skin flap and, thus, was used in the subsequent endoprosthesis surgeries described below. Next, a circumferential incision was made approximately at the level of the metacarpophalangeal joint. Sharp dissection was used to dissect the skin from the underlying musculoskeletal tissue distal to the level of the amputation at approximately 2 cm from the distal end of the tibia. A pneumatic, right-angled, 6-mm-wide osteotomy saw (micro-oscillating saw, Salvin Dental Specialties, Charlotte, NC, USA) was used to perform an osteotomy of the distal 1/3rd of the tibia at a point approximately 2-cm from the distal end of the tibia. Indocyanine green, a fluorescent dye, was injected in a T-port ear vascular catheter. The skin of the hindlimb was imaged using a fluorescence imaging machine (SPY, Stryker, Kalamazoo, MI, USA). A mock silicone foot–ankle prosthesis (16A Shore hardness, Figure 2A) was inserted into the medullary canal at the distal end of the tibia. The prosthesis length was trimmed as needed, then the dissected skin was elevated over the prosthesis and closed with suture. Indocyanine dye injection and fluorescence imaging were repeated.

### 2.2. Endoprosthesis Prototype Design

The endoprosthesis prototype design consisted of a tibial intramedullary pin and stem attached to a foot segment (Figure 1A). Since the study was focused on skin coverage, all components were fabricated as a single piece so that the ankle joint (i.e., the joint between the tibial and foot segments) was fused and rigid. We created a three-dimensional model of the endoprosthesis prototype in computer-aided design software (SolidWorks, Dassault Systèmes, Vélizy-Villacoublay, France). To improve mechanical stability at the bone–implant interface, we included eyelets for attaching biological tendons on the cranial (tibialis anterior tendon) and caudal (Achilles tendon) aspects of the stem. The ankle was fixed in a dorsiflexed angle (35 degrees for E1, 80 degrees for E2 and E3) to approximate the dorsiflexed posture of the biological ankle in rabbits during sitting. The core component of the endoprosthesis prototypes were 3-D printed in corrosion-resistant alloy 316L stainless steel by direct metal laser sintering (FS271M, Farsoon Technologies, Hunan, China). The metal component, except for the intramedullary pin and eyelets, was over-molded with biocompatible liquid silicone rubber (BIO M340, Elkem Silicones, East Brunswick, NJ, USA) to provide a soft substrate for skin.

### 2.3. Feasibility Study: Surgical Implantation of Endoprosthesis Prototypes

The rabbits were first premedicated with hydromorphone, midazolam, and lidocaine; sedated; induced into general anesthesia; and maintained by isofluorane gas vaporized in 100% O_2_. A skin flap was generated as described above for the pilot study, using an initial longitudinal incision along the lateral aspect of the hindlimb. As in the pilot study, a pneumatic, right angled, 6-mm wide osteotomy saw was used to perform an osteotomy of the distal 1/3rd of the tibia at a point approximately 2-cm from the distal end of the tibia. The tibia intramedullary canal was cleared of fat. Polymethylmethacrylate (PMMA) bone cement was injected into the intramedullary canal of the tibia through the distal (open) end. An endoprosthesis prototype was inserted in the intramedullary canal and held in place until the bone cement cured. The Achilles and tibialis cranialis biological tendons were cut at the enthesis, passed through its corresponding eyelet (noted above) on the endoprosthesis, then folded back on and sutured to itself using 3-0 PDS suture. The subcutaneous tissues and skin were elevated over the endoprosthesis, trimmed as needed, and closed using 5-0 PDS suture in a simple continuous suture pattern. The skin was closed with 3-0 PDS suture in a combination of cruciate and simple continuous sutures.

### 2.4. Post Surgery

To facilitate recovery, we administered hydromorphone to the rabbits every 6 h for at least 72 h post surgery to manage acute pain; meloxicam every 24 h for at least 7 days post surgery to manage pain and swelling; and enrofloxacin every 12 h for at least 7 days post surgery to prevent infection. Fluids (about 300 mL daily) were provided subcutaneously for 3 days post surgery. Food and water consumption were monitored daily for two weeks post surgery, and body mass was measured once per week for the duration of the study. The rabbits were euthanized either at approximately 60 days post surgery (past date of expected wound healing based on our previous study [25]) or at a humane endpoint (e.g., non-repairable skin dehiscence), whichever occurred first. After euthanasia, both hindlimbs were harvested, shaved, and fixed in 10% phosphate-buffered formalin for at least 5 days for tissue evaluation.

### 2.5. Histology

For preliminary measurement of tissue thickness, samples of skin and subcutaneous tissue (if present) were collected from E1 at the following locations on both the operated and intact contralateral limbs: the dorsal, ventral, lateral, and medial aspects of the foot; the lateral and medial aspects of the ankle; and the hindlimb shank proximal to the endoprosthesis. The formalin-fixed tissues were embedded in paraffin in tissue blocks. The blocks were sectioned, mounted on slides, routinely stained with Hematoxylin & Eosin (H & E), and cover-slipped. We measured the thickness of the skin (epidermis + dermis) and subcutaneous tissue (if present) on the medial, ventral, and lateral aspects of the endoprosthesis and foot on the operated and non-operated sides, respectively. Thickness measurements were performed using image processing software (ImageJ version 1.53, NIH, Bethesda, MD, USA). For each histology section, we measured thickness at 8 to 13 equally spaced locations along the length of the section, then computed the mean and standard deviation of the measurements.

Select tissue samples were collected for histopathological evaluation (IDEXX BioAnalytics, Inc., Columbia, MO, USA); samples of the skin and subcutaneous tissues (if present) were taken from the lateral (E1, E3) or ventral (E2) aspects of the endoprosthetic (operated side) or biological (non-operated side) foot. 

## 3. Results

In the pilot study, fluorescence imaging indicated adequate blood perfusion throughout the dissected and reconstructed skin flaps in rabbits N1 and N2. Qualitatively, blood flow appeared better with the lateral longitudinal incision as compared with the cranial longitudinal incision (Figure 2). This result motivated the decision to use a lateral longitudinal incision to create the skin flap for the endoprosthesis prototypes in rabbits E0–E3.

**Figure 3:**
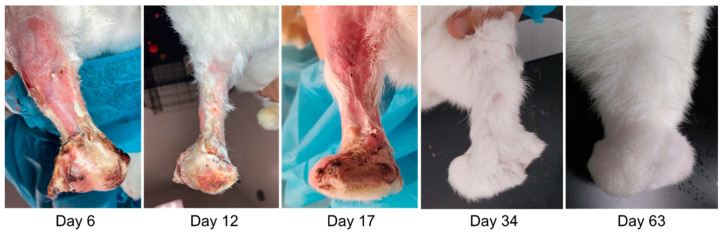
Progression of skin healing and fur regrowth up to 63 days post surgery (study endpoint) in rabbit E1.

Rabbits E1–E3 successfully recovered from surgery and maintained skin integrity up to the pre-defined study endpoint at 60 days post surgery. The incisions fully healed by about 30 days post surgery, and all limbs had substantial fur regrowth by about 60 days post surgery (Figure 3). Upon manipulation, the endoprostheses felt mechanically stable within the tibia bone at the study endpoint.

**Figure 4:**
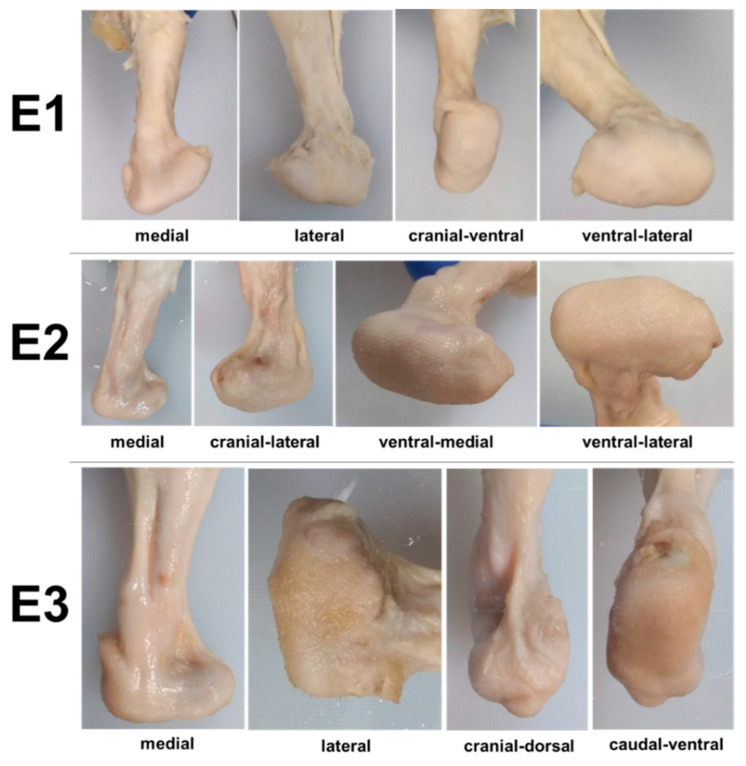
Photographs of the formalin-fixed skin with fur removed from rabbits E1–E3. The skin surrounding the endoprostheses appeared normal.

**Figure 5:**
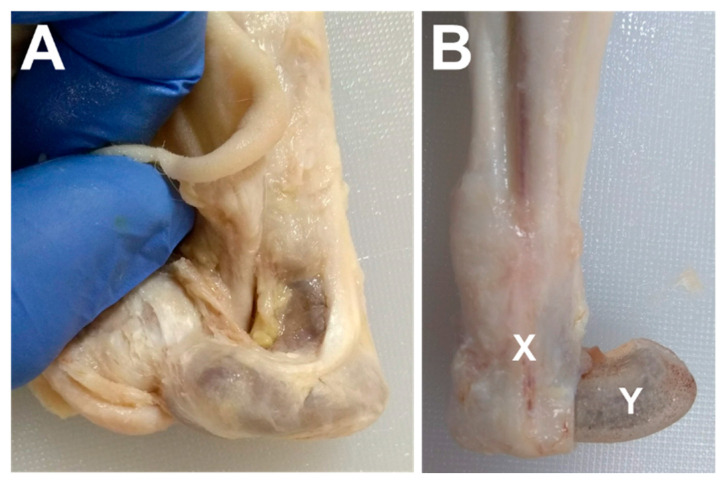
Fibrous encapsulation of endoprosthesis on (**A**) rabbit E2 and (**B**) rabbit E3, with “X” indicating the in situ encapsulation and “Y” indicating the endoprosthesis with the encapsulation removed. All three rabbits exhibited encapsulation around the endoprosthesis.

Ex vivo gross examination revealed that the skin surrounding the endoprosthesis was contiguous and appeared healthy, with no swelling, discoloration, or other signs of pathology (Figure 4). The skin appeared to conform to the shape of the endoprosthesis. Dissection of the skin revealed a substantial fibrous capsule surrounding the endoprosthesis in all three rabbits (Figure 5).

**Figure 6:**
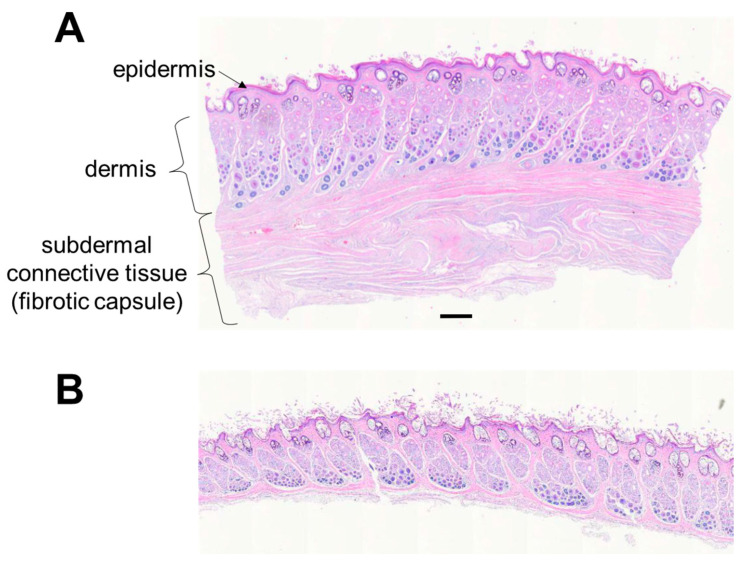
Histology of tissues (rabbit E2) from the ventral aspect of (**A**) the endoprosthetic foot in the operated limb and (**B**) the biological foot in the non-operated limb. Bar = 0.64 mm.

Histologically, the structure and thickness of the epidermis and dermis were similar between the operated and non-operated limbs in E1 and E3 (Figure 6). There was mild epidermal and dermal thickening on the dorsal aspect of the endoprosthesis in E2. The approximate number of cell layers and thickness of the epidermis in E2 were greater in the operated limb (10 cell layers, 0.075 mm) than in the non-operated limb (3–5 cell layers, 0.015 mm), respectively. The dermis in E2 was about 2 mm thick in the operated limb and 1–1.5 mm thick in the non-operated limb.

**Figure 7:**
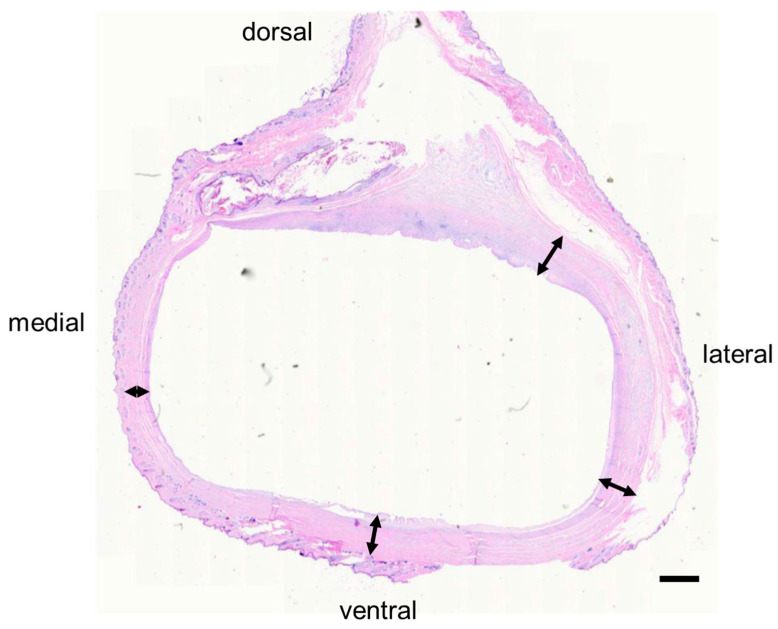
Histology of tissues (rabbit E1) surrounding the endoprosthetic foot, which previously occupied the oval-shaped void in the center. Arrows indicate sub-cutaneous tissue.

Unlike the non-operated limb, the operated limb had a substantial layer of subdermal connective tissue representing the fibrous capsule observed grossly. The connective tissue consisted of multiple longitudinal and more-superficial circumferential bands of collagenous fibrovascular tissues; these surrounded deeper, less-mature areas of fibroplasia that encircled and apposed the endoprosthesis (Figure 7). The subcutaneous connective tissue layer was thicker than the skin in rabbit E1 (Table 2).

## 4. Discussion

Our in vivo study successfully demonstrated the feasibility of implanting a foot–ankle endoprosthesis completely within living skin at the distal end of a residual limb in a rabbit model of transtibial amputation. The foot–ankle endoprosthesis required a larger skin flap than the device, a 2-cm-long unjointed stem endoprosthesis, that we tested in the same in vivo model in a previous study [25]. In both studies, most of the fur had regrown by about 60 days post surgery, one indicator that the function of the skin was preserved. Our current and previous study focused on the feasibility of covering endoprostheses in living skin. Therefore, unlike the biological ankle joint, the foot–ankle endoprosthesis had a rigid (i.e., fixed) ankle joint. However, a key motivation for implanting prosthetic limbs within skin is to facilitate direct, physical attachment of muscles to prostheses across prosthetic joints. Doing so is expected to permit direct neuromuscular control of prosthesis movements. To test the concept of direct muscular actuation and control of endoprosthesis movements, in future studies, we plan to fabricate foot–ankle endoprostheses with a functional jointed ankle. 

Both gross examination and histology results preliminarily indicated that the endoprosthesis did not adversely affect the overlying skin. At the study endpoint, about 60 days post surgery, the shaved skin surrounding the endoprostheses appeared normal. The thickness, structure, and appearance of histology samples of the epidermal and dermal layers of the skin were similar between the operated and non-operated limbs; these layers exhibited only mild thickening in histology samples from E2. 

That a fibrous capsule formed on the endoprosthesis was not surprising. Fibrous encapsulation is the end result of a process referred to as a foreign body response, which has been well described [26]. The characteristics of the fibrous capsule, specifically the subdermal connective tissue, were similar between our study and other studies of subdermal implants [27]. Fibrotic tissue is much stiffer than skin [28] and, thus, could reduce the potential range of motion of jointed endoprostheses we plan to test in future studies. Some factors, such as the use of soft silicone and administration of integrin inhibitors [27], have been shown to suppress fibrotic encapsulation of subdermal implants. We will investigate the effectiveness of such factors for suppressing fibrosis with our next-generation endoprosthesis designs in future studies. 

The proposed endoprosthetic limb is intended primarily for patients with amputation but may be challenging to implement based on the cause of amputation and patient’s health status. For example, in our feasibility study, the amputation and prosthesis implantation were performed in the same surgery; doing so allowed us to create an “ideal” skin flap from the native skin that surrounded the amputated foot and ankle; thus, the skin flap maintained vascular continuity with the proximal skin. However, for people with traumatic or congenital amputation, it may not be possible to salvage skin from the amputated portion of the limb to cover a prosthesis. In such cases, other sources of skin would be needed. A conventional option would be an autologous skin graft created with tissue expanders [29,30]. Another potential option is tissue-engineered skin, which is approved for select clinical indications [31] but would need further development and testing for endoprostheses. 

An endoprosthesis may also be challenging to implement in patients with dysvascular disease, which is identified as the cause of as many as 82% of all amputations [32]. Such amputations are often a result of skin problems, such as ulcers, that fail to heal due to poor circulation and neuropathy associated with dysvascular disease [33]. These complications may prevent adequate circulation and wound healing in the skin flap or graft used to cover an endoprosthesis. Thus, endoprostheses may be more feasible for patients with traumatic, cancer-related, or congenital amputations who are expected to have healthier circulation and nerve function.

In some respects, the proposed endoprosthesis is similar to those used for extensive skeletal reconstruction in orthopedic oncology patients. For example, both types of endoprostheses are intended to replace large sections of, and potentially even entire, bones. The successful clinical use of large endoprostheses [20,21] provides strong evidence for the potential clinical feasibility of our proposed endoprosthetic limbs for people with amputation. However, as noted in the Introduction, there are notable differences in tissue interface and anatomical placement between the existing and proposed endoprostheses. These differences underlie the need for the research reported here.

The design of the endoprosthesis prototype was sufficient for our feasibility study but could be further optimized by leveraging techniques from standard and state-of-the-art orthopedic implants. For example, though stainless steel is used in some orthopedic implants, titanium and cobalt-chromium alloys have superior strength-to-weight ratios and corrosion resistance [34]. Bone cement is a relatively simple and conventional fixation material [35] that maintained structural stability of the endoprosthesis relative to the residual tibia bone in our short-term study. More advanced methods involving porous materials and coating could potentially achieve better osseointegration without bone cement [36,37,38]. 

We anticipate that, besides permitting direct muscle attachment, implanting limb prostheses within living skin will have other benefits for patients. Specifically, convenience, comfort, health, cosmesis, and function could be superior with endoprostheses than with traditional external prostheses. For example, patients will not have to don/doff the endoprosthesis, even in extreme environments (e.g., underwater) that may damage external electromechanical prostheses. There will be less risk of discomfort and skin problems, such as blisters or sweating, associated with traditional sockets worn over the skin [39,40]. Compared to osseointegrated sockets that protrude through the skin, there will be less risk of infection [41]. By being fully enclosed within skin, an endoprosthesis would appear as a more natural extension of the body than external prostheses. Patients may also experience natural somatosensation (e.g., touch) through the overlying skin, which could improve overall functional ability [42]. The potential collective effect of these benefits is that patients will have a greater sense of limb embodiment and, thus, better overall satisfaction and quality of life with endoprostheses [43].

There were several limitations of our study. First, both the vascular imaging pilot study and the endoprosthesis feasibility study had relatively low sample sizes. The number of animals was sufficient to demonstrate feasibility, though future studies should include more animals to better account for potential inter-subject variability in outcomes. Second, the duration of the feasibility study was relatively short (60 days); future studies should be longer to identify potential adverse skin changes that could occur beyond 60 days and with active use of the limb for functional tasks (e.g., locomotion). Third, we measured thickness of tissues from only one rabbit (E1); this was because we inadvertently dissected the skin from most of the subcutaneous tissue in rabbits E2 and E3, which prevented us from collecting histology samples that included both skin and subcutaneous tissue at all locations of interest (Table 2). In future studies, we will revise our dissection method to ensure that we can perform histology and thickness measurements for all animals. Finally, our study was performed in rabbits, which exhibit similar wound healing characteristics [44]. However, there are likely some other relevant differences between rabbits and humans that may limit the applicability of our results to human patients. Therefore, human clinical trials will ultimately be needed to achieve clinical translation of our proposed endoprosthesis device and approach.

## 5. Conclusions

In conclusion, our study demonstrated that it is feasible to implant a rigid foot–ankle endoprosthesis prototype within living skin in a rabbit model of transtibial amputation. The success of the endoprosthesis was enabled, in part, by the vascular imaging pilot study to determine the size of the skin flap and endoprosthesis foot segment. Our results and surgical experience will inform a future study to test a foot–ankle endoprosthesis with a rotatable ankle joint in our in vivo model. 

## Figures and Tables

**Table 1:** Information about rabbits used in the study.

Procedure	Rabbit	Age at Surgery	Body Mass at Surgery (kg)	Euthanasia, Days Post Surgery
skin vascular imaging (non-survival)	N1	34 weeks, 2 days	4.18	-
	N2	34 weeks, 2 days	4.20	-
rigid foot–ankle endoprosthesis	E0 *	18 weeks, 5 days	3.18	0
	E1	18 weeks, 5 days	3.57	63
	E2	18 weeks, 4 days	4.30	64
	E3	18 weeks, 4 days	3.16	64

* Died < 1 h after surgery due to suspected anesthetic complications.

**Table 2:** Thickness (mm) of tissues surrounding the medial, ventral, and lateral aspects of either the endoprosthetic (operated) or biological (non-operated) foot in rabbit E1 (n = 1). Each value (mean ± standard deviation) was computed from 8 to 13 measurements from a single histology section.

	**Medial**		**Ventral**		**Lateral**	
**Tissue**	**Operated**	**Non-** **Operated**	**Operated**	**Non-** **Operated**	**Operated**	**Non-** **Operated**
skin (dermis + epidermis)	0.43 ± 0.06	0.34 ± 0.05	0.80 ± 0.06	1.71 ± 0.04	0.76 ± 0.08	0.93 ± 0.07
subcutaneous tissue	0.85 ± 0.03	-	1.47 ± 0.06	-	1.42 ± 0.08	-

## Data Availability

Not applicable.
